# Driving Habits of Older Adults in Massachusetts: Variations by Sex, Age, Race, Income, Housing Density, and Health

**DOI:** 10.1093/geroni/igab046.097

**Published:** 2021-12-17

**Authors:** Wenjun Li, Elizabeth ProcterGray, Kevin Kane, Jie Cheng, Anthony Clarke

**Affiliations:** University of Massachusetts Lowell, Lowell, Massachusetts, United States

## Abstract

Maintaining ability to drive is critical to independent living among older adults residing in suburban and rural communities. We administrated structured questionnaire about driving behaviors to 370 persons age 65 and older living in Central Massachusetts between 2018 and 2020. Of them, 307 were active drivers. Driving in the past year was strongly associated with being male, White race, higher income, non-urban resident, and good-to-excellent health. Advancing age was associated with lower frequency of driving, less miles driven, lower percentage of the day spent in transportation. Men and women drove with nearly equal frequency (~26 days/month), but men drove significantly more miles. Non-White drivers were significantly more likely to avoid driving out of town or in difficult conditions, even after controlling for age, sex, income, and density of residential area. In conclusion, driving behaviors differed significantly by age, sex, income, race, and housing density. Further investigation is warranted.

